# Heat-activated intravenous liposomal doxorubicin combined with mitomycin C hyperthermic intravesical chemotherapy (HIVEC) for treatment of bladder cancer

**DOI:** 10.1080/02656736.2026.2615462

**Published:** 2026-02-03

**Authors:** Andrew S. Mikhail, Sandeep Gurram, Vladimir A. Valera, Dieter Haemmerich, Nikhil Gopal, Ivane Bakhutashvili, William Richardson, Cody J. Peer, Matthew F. Starost, Charles Hesswani, Keith T. Schmidt, William D. Figg, John W. Karanian, William F. Pritchard, Bradford J. Wood

**Affiliations:** a Center for Interventional Oncology, Department of Radiology and Imaging Sciences, Clinical Center, National Institutes of Health, Bethesda, Maryland, USA; b Urologic Oncology Branch, National Cancer Institute, National Institutes of Health, Bethesda, Maryland, USA; c Department of Pediatrics, Medical University of South Carolina, Charleston, South Carolina, USA; d Clinical Pharmacology Program, National Cancer Institute, National Institutes of Health, Bethesda, Maryland, USA; e Division of Veterinary Resources, National Institutes of Health, Bethesda, Maryland, USA

**Keywords:** Urinary bladder neoplasms, liposomes, hyperthermia, mitomycin, doxorubicin

## Abstract

**Purpose:**

To evaluate the safety and pharmacokinetics of lyso-thermosensitive liposomal doxorubicin (LTLD, ThermoDox®) combined with mitomycin C (MMC) hyperthermic intravesical chemotherapy (HIVEC) as a potential treatment for non-muscle invasive bladder cancer.

**Materials and methods:**

Four healthy swine received a 30-minute intravenous infusion of LTLD in combination with HIVEC, consisting of one hour of warm MMC bladder recirculation. Full-thickness bladder wall specimens were surgically resected within 30 min of HIVEC completion for histologic analysis and assessment of doxorubicin (DOX) intrinsic fluorescence in cross sections. DOX and MMC concentrations were also measured in bladder specimens and in serial blood samples up to 4 h by LC/MS. Swine were monitored for 28 days for signs of toxicity and were weighed weekly. Complete blood counts were performed at 4 h and 28 days. Cystoscopy was performed at 28 days, and bladder tissue was collected for histology and quantification of drug concentrations.

**Results:**

Mean DOX concentration in the bladder wall 30 min after treatment was 13.6 ± 4.67 μg/g, and DOX fluorescence was visible in all layers. Plasma DOX C_max_ and AUC_0−∞_ were 16.4 ± 3.46 μg/mL and 23.2 ± 5.50 μg/mL · h, respectively. MMC was not detected in the bladder wall or blood above the lower limit of detection (20 ng/mL). The treatment was well tolerated, with no treatment-related adverse events and no weight loss during the 28-day observation. On day 28, cystoscopy demonstrated normal-appearing bladder lumen, and some vacuolated cells were observed on bladder wall histology.

**Conclusions:**

Intravenous LTLD combined with MMC HIVEC was feasible and well tolerated in swine.

## GRAPHICAL ABSTRACT

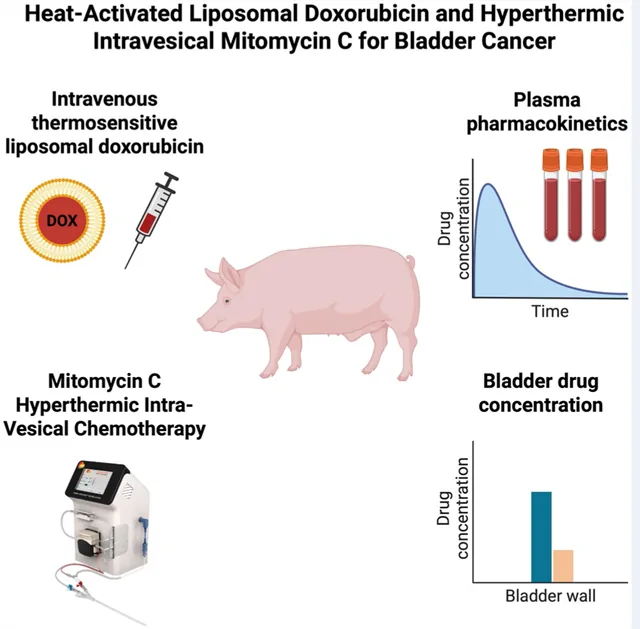

## Introduction

Urinary bladder cancer is the sixth most prevalent cancer in the United States and the fourth most common in men [1]. Non-muscle-invasive bladder cancer (NMIBC) accounts for 75% of all bladder cancer and is treated by transurethral resection of the bladder (TURBT), with or without intravesical therapy, depending on tumor risk stratification. The standard of care for high-risk NMIBC consists of TURBT followed by induction and maintenance courses of Bacillus Calmette-Guérin (BCG) intravesical therapy. For BCG-unresponsive patients, radical cystectomy is the standard of care but is associated with 90-day complication and mortality rates of 64–80% and 2.7–4.7%, respectively [2–4]. Alternative therapies are needed for patients who are not eligible for surgery or desire more conservative, bladder-sparing treatments.

Several intravesical and systemic agents are available for BCG-unresponsive patients; however, these agents have limited long-term efficacy and may cause significant side effects [5]. Intravesical drug delivery increases local drug concentrations and reduces systemic exposure and toxicity compared to systemic administration. However, the low permeability of the urothelium restricts drug uptake and penetration into the bladder wall, making intravesical therapy most suitable for superficial tumors [6–8]. Hyperthermic intravesical chemotherapy (HIVEC) involves recirculating a warm (42–44 °C) chemotherapy solution inside the bladder. Potential benefits of hyperthermia include increased tissue permeability to intravesical drugs [9], chemosensitization of bladder cancer cells [10], and stimulation of immunogenic effects [11].

Lyso-thermosensitive liposomal doxorubicin (LTLD) (ThermoDox®) consists of liposome-encapsulated doxorubicin (DOX) that is released upon exposure to mild hyperthermic temperatures (39–42 °C) [12,13]. Preclinical studies have demonstrated the use of temperature-responsive liposomes to localize high concentrations of DOX in the bladder wall following systemic delivery and intravesical mild hyperthermia [14,15] and to improve tumor control [16]. Combining systemic LTLD with intravesical delivery of hyperthermic mitomycin C (MMC) may provide additional benefits including enhanced tumor drug coverage and potential synergistic therapeutic effects.

The objective of this study was to evaluate the feasibility and safety of combined therapy with LTLD and HIVEC in a healthy swine model and measure plasma pharmacokinetics and bladder wall uptake of DOX and MMC.

## Materials and methods

### Computational modeling of tissue heating and drug delivery in the bladder wall

Computer models were developed to simulate tissue heating and drug delivery, as previously described, with some modifications related to the heat transfer model [14,17]. An axisymmetric 2D model was created where a 4.2-mm thick bladder section was modeled, including 0.71 mm mucosa (urothelium þ lamina propria). We assumed heterogenous perfusion with higher perfusion in the mucosa, as in our prior study [14]. The drug delivery model considered temperature-dependent intravascular release of DOX, extravasation of released DOX in heated tissues, and cellular DOX uptake as explained in detail in prior studies [14,17]. The temperature-dependent liposomal release used in the model was based on prior *in vitro* measurements [18]. The heat transfer model assumed surface tissue heating from heated water at 43 °C, which was simulated by applying a convective heat transfer coefficient of *h* = 100 W/(K m^2^) to the surface [14]. In addition, the model simulated heat conduction into the bladder wall, and cooling due to blood perfusion that was modeled based on Pennes’ Bioheat Transfer Equation (BHTE) [19]. The objective was to determine the optimal timing of the LTLD and HIVEC infusions to maximize DOX deposition in the serosa. We assumed a 30-min infusion in the computer model, during which time LTLD were continuously added to the plasma compartment of the model. We performed multiple simulations where the timing between infusion and heating was varied to determine the optimal timing that resulted in highest drug concentration in the serosa. This provided a conservative estimate of DOX deposition throughout the bladder wall, given the expected limitations of warming the serosa due to its distance from the heat source.

### Animal model

All animal procedures were approved by the Institutional Animal Care and Use Committee and performed in accordance with applicable federal regulations. Four female yorkshire swine (18–20 weeks old, 62–72 kg; Oak Hill Genetics, Ewing, IL) were studied; this sample size was sufficient to identify safety concerns in a single-dose feasibility study. Due to a known risk of anaphylactoid reaction to liposomal formulations, swine were premedicated as follows. Dexamethasone (0.12 mg/kg, IM) was administered twice daily for 48 h prior to LTLD delivery. The day before LTLD infusion, famotidine (0.5 mg/kg, IM) was administered. Dexamethasone (0.12 mg/kg, IM), famotidine (0.5 mg/kg, IM), and diphenhydramine (2 mg/kg, IM) were given 1.5–3 h before LTLD infusion. Anesthetic induction was performed with IV propofol (1 mg/kg) following sedation with intramuscular ketamine (25 mg/kg), midazolam (0.5 mg/kg), and glycopyrrolate (0.01 mg/kg). Animals were intubated and a surgical plane of anesthesia was maintained with 100% oxygen and isoflurane (1–5%). Penicillin G (3,400 units/kg IM) was administered. Bilateral jugular venous catheters were inserted under ultrasound for blood collection (left jugular vein) and LTLD infusion (right jugular vein). Cystoscopy was performed and the bladder was catheterized. Meloxicam (0.3 mg/kg, IV) was given 10 min before infusion of LTLD. At the conclusion of the treatment, including collection of bladder tissue, blood samples, and wound closure, the swine were recovered, housed for 28 days, and weighed weekly. At day 28, cystoscopy was performed under general anesthesia followed by euthanasia by intravenous administration of Beuthanasia-D (pentobarbital sodium 390 mg/mL and phenytoin sodium 50 mg/mL).

### Treatment protocol

**Figure 1.**
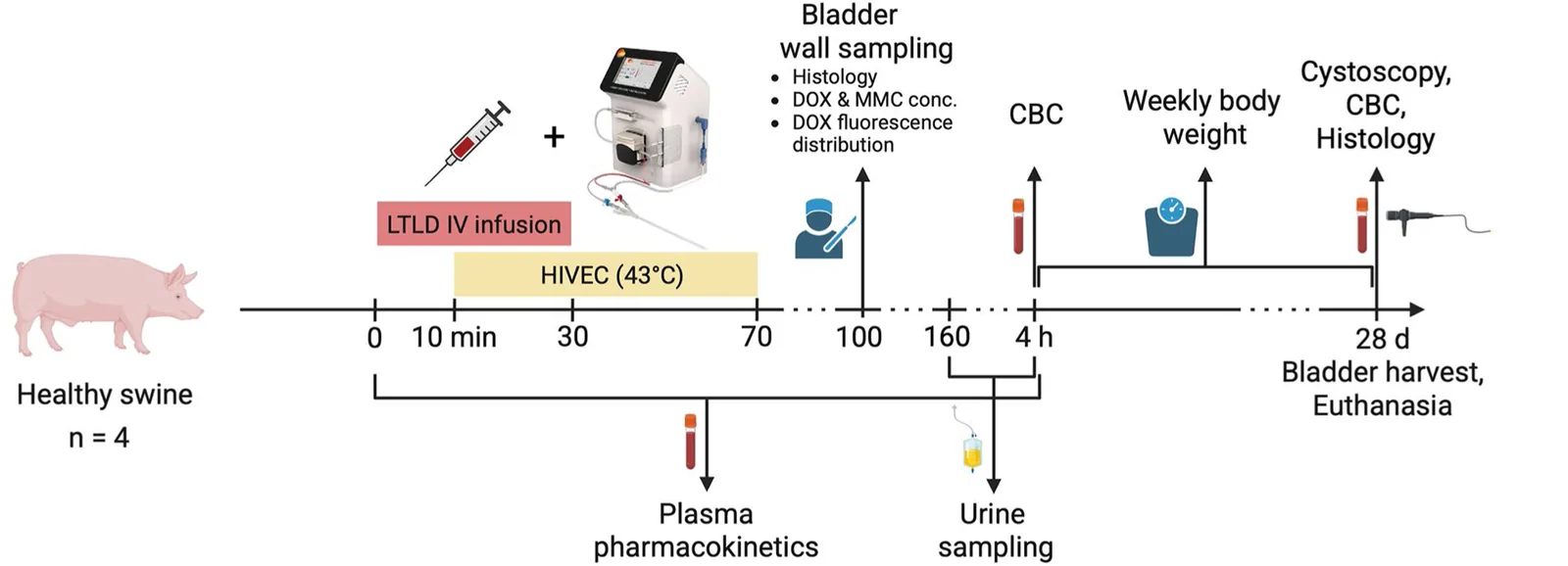
Experimental outline. LTLD: lyso-thermosensitive liposomal doxorubicin; HIVEC: Hyperthermic IntraVesical Chemotherapy; CBC: complete blood count; DOX: doxorubicin; MMC: mitomycin C.

LTLD (0.7 mg/kg) (ThermoDox®, Imunon, formerly Celsion Corp., Lawrenceville, NJ), was administered as a 30-min IV infusion (Figure 1). This dose corresponds to approximately 40 mg/m^2^ when calculated by allometric scaling using pig body surface area (BSA, m^2^), according to the formula: BSA = 0.0734 * body weight (kg)^0.656^ [20]. HIVEC was performed using a Bladder Recirculation System (Combat BRS, Combat Medical, St Albans, UK) in accordance with the manufacturer’s recommendations. The tubing was primed with 40 ml sterile water and connected to a flexible 16 Fr 3-way urinary catheter containing a thermocouple at the tip. The water was recirculated through the system, excluding the bladder, for several minutes to partially warm the fluid. The treatment solution, 40 mg of MMC (Accord, UK) with 80 mg mannitol dissolved in 40 ml sterile water, was instilled into the bladder, which had been drained of urine. Recirculation through the system and bladder began 7 min after initiation of the LTLD IV infusion, ensuring that the MMC solution, monitored continuously by an in-line temperature probe in the inflow channel, reached the target operating temperature of 43 °C at the start of HIVEC after an approximate 3-min heat-up period. This achieved the optimal 10-min offset between the beginning of LTLD infusion and HIVEC for maximum DOX deposition in the bladder, as determined by computational modeling. HIVEC was administered for 1 h. After completion of HIVEC, the MMC solution was withdrawn using a syringe attached to the catheter, and the bladder was rinsed with saline.

### Blood, urine, and tissue collection and analysis

A lower midline laparotomy was performed to expose the bladder, which was released from its surrounding attachments. Two 2 × 2 cm full-thickness bladder samples were collected from the anterior and posterior walls of the bladder within 30 min of HIVEC completion. Care was taken to avoid sampling the bladder trigone and urethra. One half of each sample was frozen in liquid nitrogen for subsequent analysis of drug content and the other half was fixed in formalin for histopathology and microscopy of DOX intrinsic fluorescence. The bladder was repaired in a watertight fashion, and the abdominal incision was closed with instillation of 10 ml Penicillin G (3 million units) across the layers of the wound closure. Following surgical closure of the bladder, urine was continuously drained via Foley catheter into a collection bag, which was emptied at 1, 1.5, and 2 h, with measurement of interval urine volume and collection of an aliquot for drug quantification. Blood samples were collected at the start of the LTLD infusion and then at regular time points for up to 4 h. Blood samples were centrifuged, and the plasma was collected and frozen for pharmacokinetic analysis. Complete blood counts were performed using blood samples collected immediately before treatment, 4 h after commencement of LTLD infusion, and at 28 days post-treatment. Immediately following euthanasia on day 28, a cystectomy was performed and samples were collected from the anterior and posterior walls of the bladder, along with samples of the urethra and the ventral rectum adjacent to the bladder. Frozen tissues were homogenized, and DOX and MMC were extracted and quantified by LC/MS. DOX and MMC in plasma and urine were also quantified by LC/MS. The lower limit of quantification and lower limit of detection for MMC were 30 and 20 ng/mL, respectively, and 300 and 100 ng/mL, respectively, for DOX.

### Bladder histology and doxorubicin tissue distribution

Specimens collected from the bladder immediately after HIVEC and 28 days post-treatment were fixed in 10% formalin, processed, and embedded in paraffin. Sections, 5 μm thick, were stained with hematoxylin and eosin, and assessed by a veterinary pathologist. Images of DOX intrinsic fluorescence in unstained sections were captured using an Olympus VS200 (Tokyo, Japan) (excitation: 468 nm, emission: 635 nm) and 20× objective lens and were pseudo-colored red.

### Pharmacokinetic analysis

A non-compartmental analysis was used to determine pharmacokinetic parameters. Maximum plasma concentration (C_max_) and time to C_max_ (T_max_) were observed values. First-order elimination rate constant and terminal exponential half-life values were calculated from the slope of the plasma concentration-time curves following log-linear regression of terminal exponential phase data points. The area under the concentration-time curve (AUC_0−∞_) was calculated from zero to the time of the last measurable plasma concentration, where zero represented the start of the drug infusion, using the trapezoid method; extrapolation to infinity was calculated by dividing the last measurable plasma concentration by the terminal exponential rate constant. Clearance (CL) was calculated by dividing the dose by AUC_0−∞_.

### Statistics

A mixed effects ANOVA was performed using GraphPad Prism version 9.0.0 (GraphPad Software, San Diego, CA, USA) to compare blood counts at different timepoints. The amount of drug in bladder tissues from different locations was compared by paired *t*-test.

## Results

### Combination therapy with LTLD and HIVEC

**Figure 2.**
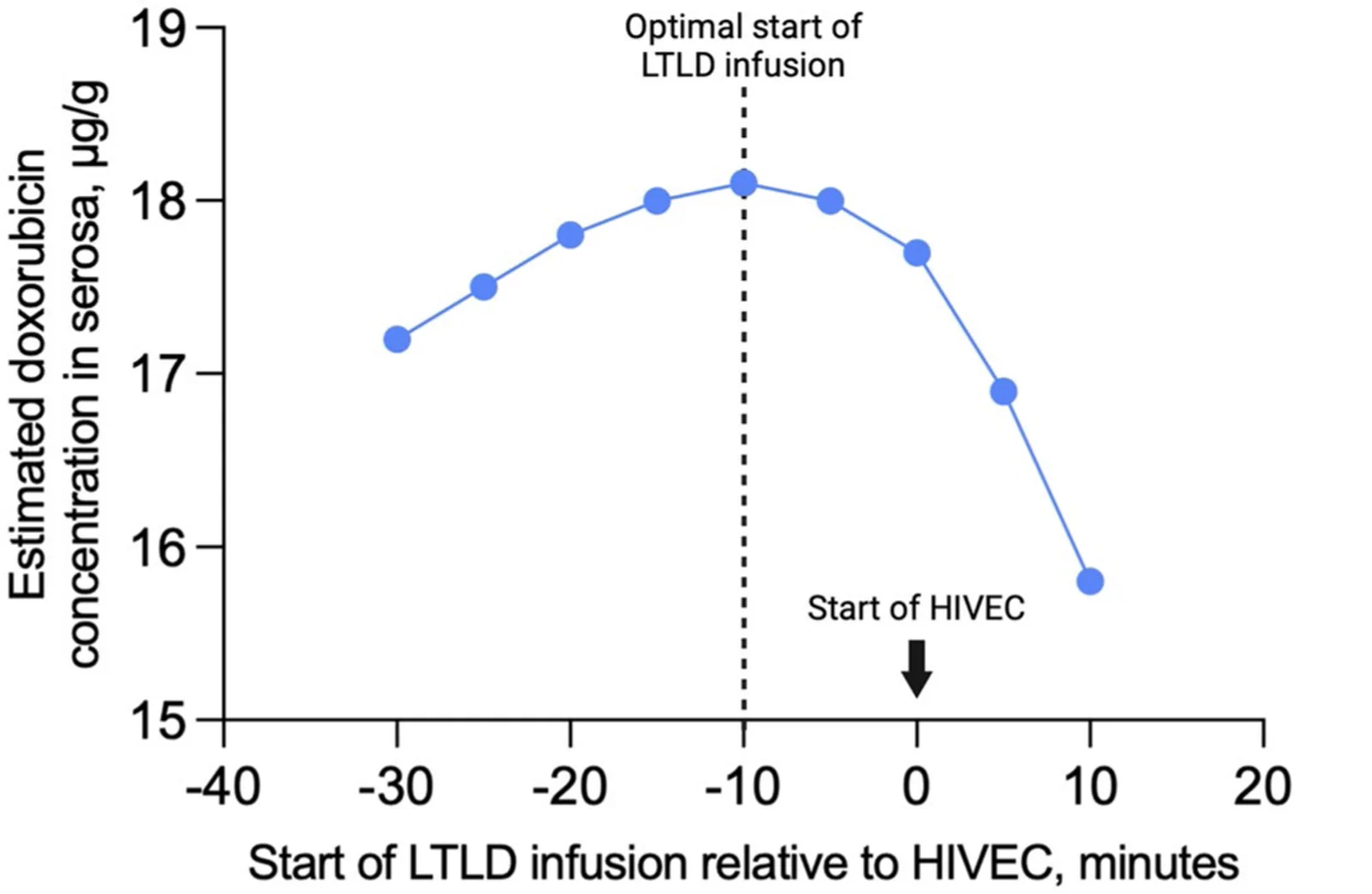
Computational modeling of the optimal timing offset for LTLD delivery relative to HIVEC. The plot shows estimated doxorubicin concentrations in the bladder serosa for a simulated 30-min LTLD infusion at various offset times between the start of LTLD infusion and the start of HIVEC (*t* = 0). Initiating LTLD infusion 10 min before HIVEC (−10 min on the x-axis) was predicted to maximize doxorubicin concentration in the bladder wall.

Computational modeling predicted that initiating LTLD infusion 10 min prior to the start of HIVEC would maximize DOX deposition in the bladder wall (Figure 2).

**Figure 3.**
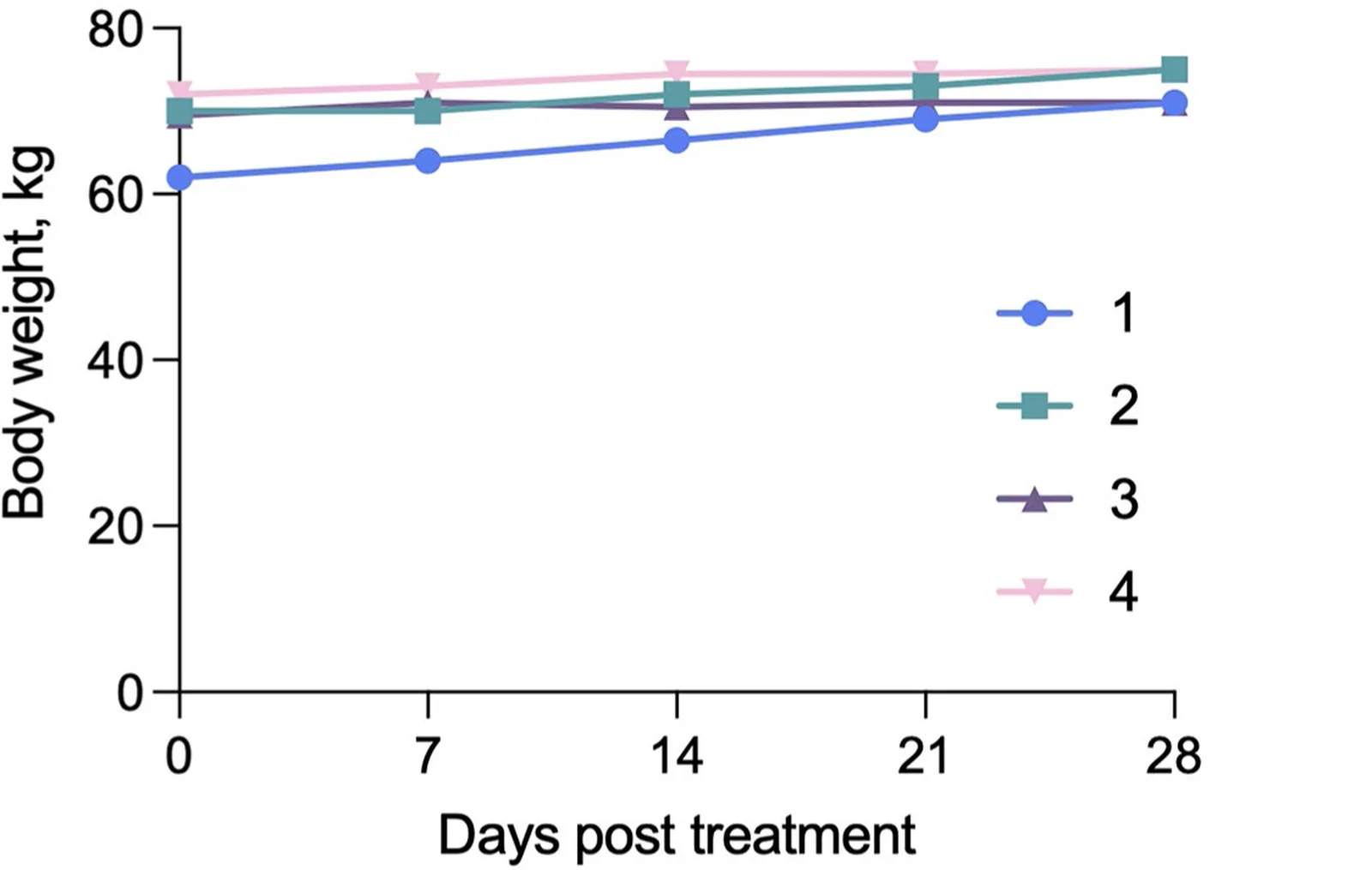
Weight change of swine following treatment. Day 0 represents the baseline weight measured prior to treatment.

**Figure 4.**
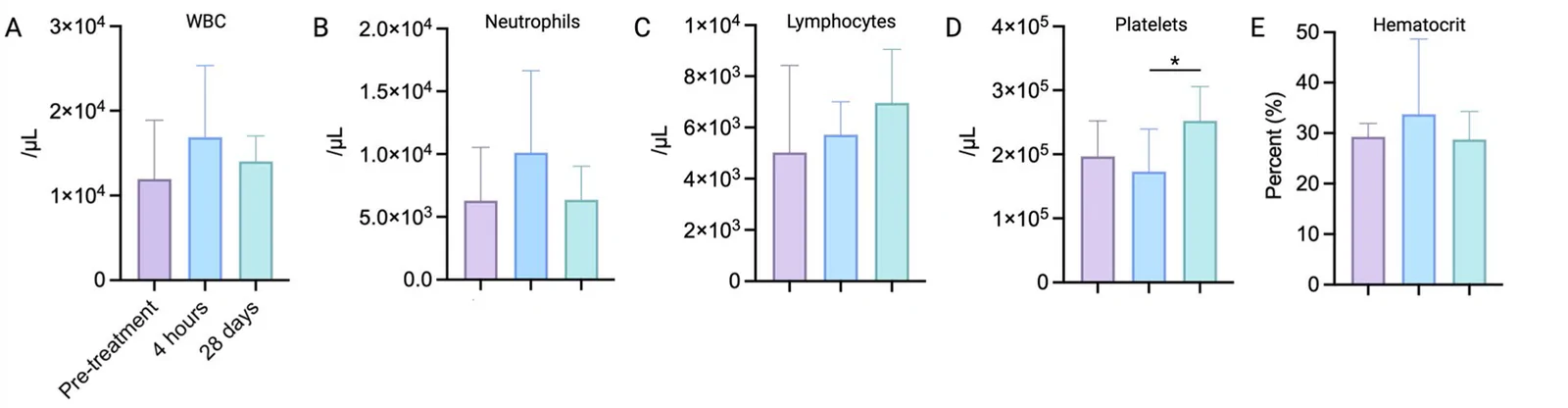
Complete blood count including A) white blood cells (WBC), B) neutrophils, C) lymphocytes, D) platelets, and E) hematocrit measured before treatment, at 4 h after commencement of LTLD infusion, and 28 days post-treatment. *indicates *p* < 0.05.

The combination treatment was well tolerated, with no adverse effects or weight loss observed during the 28-day post-treatment observation period (Figure 3). No significant differences were detected in white blood cell, neutrophil, lymphocyte, or hematocrit counts at any time point (baseline, 4 h, and 28 days). Platelet counts differed between 4-h and 28-day time points but remained within the normal range (Figure 4) [21]. One swine developed an incisional hernia with a portion of the bladder herniating through the fascial defect. Since the swine was clinically unaffected, this was managed conservatively without further intervention.

### Cystoscopy and histology of the bladder

**Figure 5.**
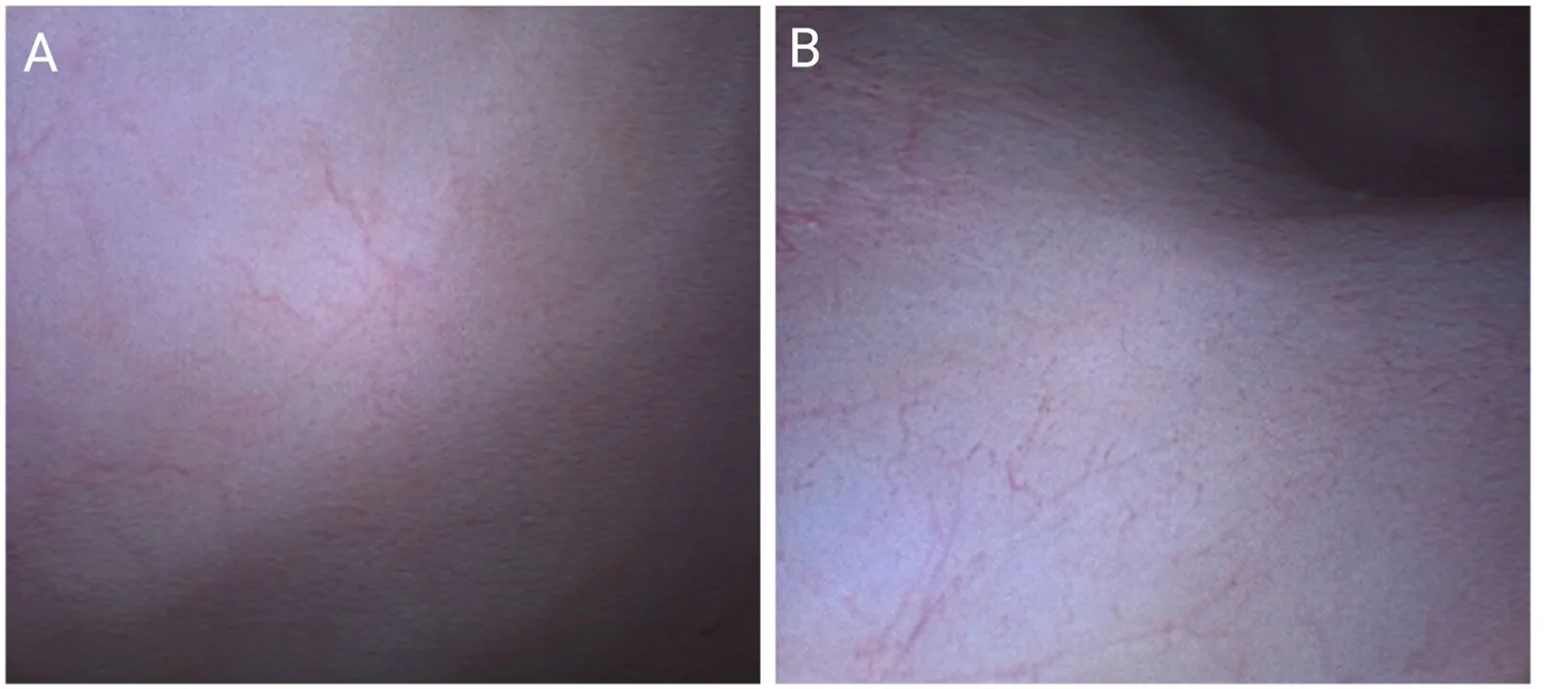
Cystoscopic images of the bladder lumen A) before and B) 28 days after treatment, without perceptible macroscopic differences.

**Figure 6.**
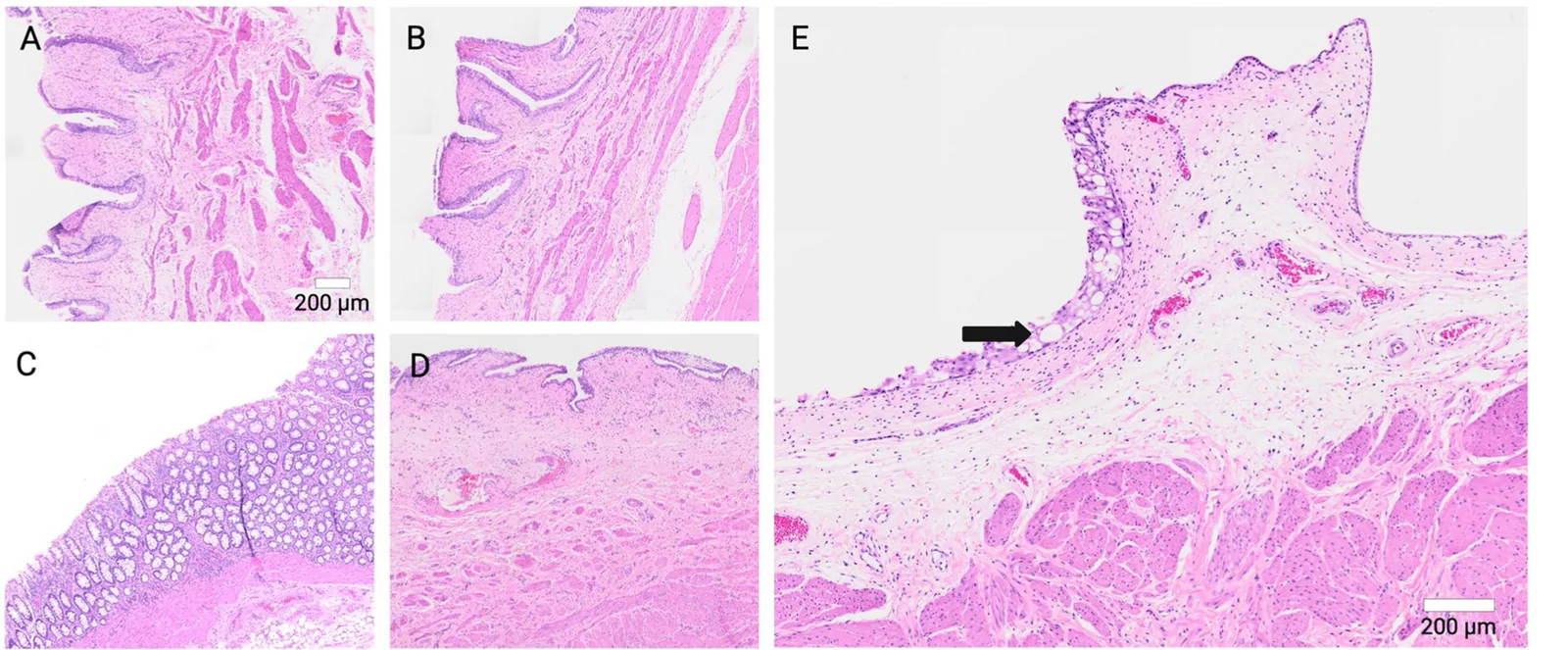
Day 0 (post treatment) and day 28 histology specimens stained with hematoxylin and eosin. A) Bladder wall after treatment on day 0. B) bladder wall; C) rectum; and D) urethra on day 28. The arrow in (E) indicates vacuolated cells in the bladder mucosa. The scale bar represents 200 μm. All images are at the same scale.

At cystoscopy, the bladder mucosa appeared normal and free of erythema or other treatment-related sequalae at 28 days post-treatment (Figure 5). Histological analysis of the bladder wall on day 0 post treatment and day 28, as well as the urethra on day 28, revealed normal tissue architecture without evidence of thermal injury (Figure 6). Vacuolated cells were observed in bladder samples from two of the four swine on day 28. A specimen taken from the rectum adjacent to the bladder was also normal.

### Drug concentrations in the bladder wall

**Figure 7.**
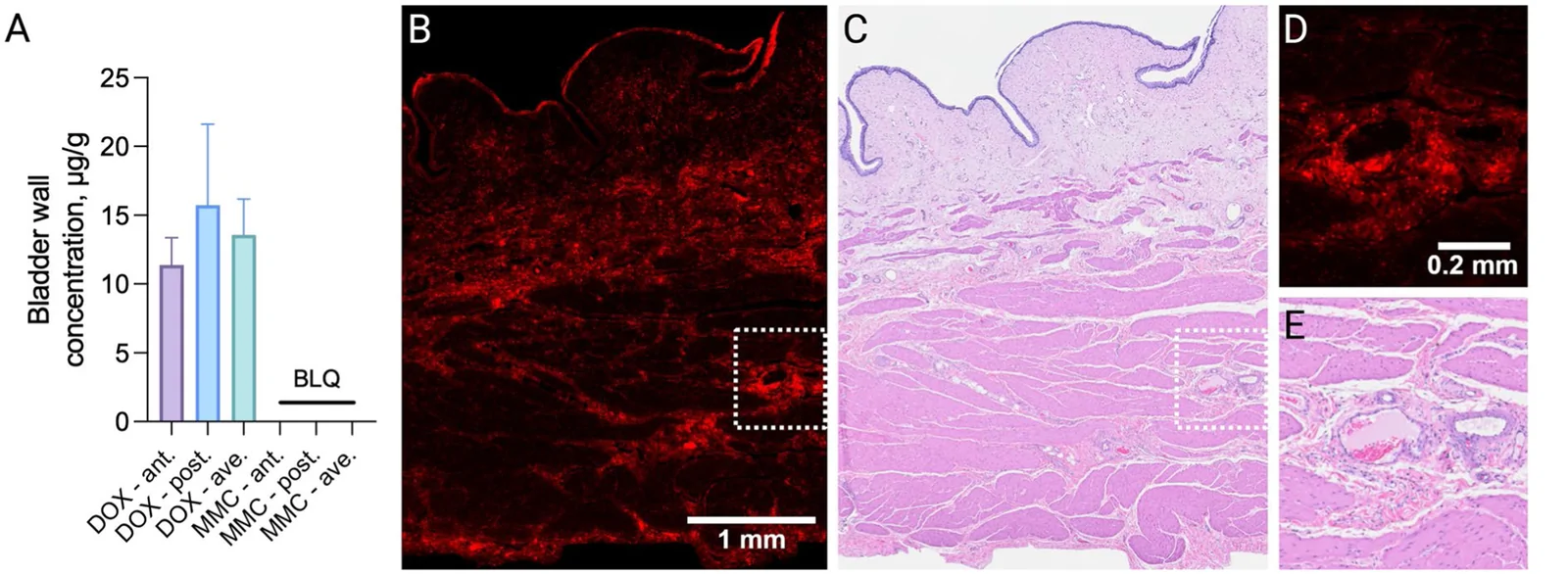
Drug accumulation and distribution in the bladder wall 30 min after completion of HIVEC and 70 min after completion of LTLD infusion. A) Drug concentrations in the bladder wall. Error bars represent SD. B) Doxorubicin fluorescence (red) in a cross-section of the bladder. C) Cross-section of bladder wall corresponding to the section in B stained with hematoxylin and eosin. The mucosa is at the top and serosa at the bottom of the images in B and C. D) and E) are magnified images corresponding to the dashed boxes in B and C, respectively, showing DOX fluorescence (D, red) within connective tissue surrounding blood vessels in the bladder wall’s middle circular muscle layer (E). DOX: doxorubicin; MMC: mitomycin C; ant.: anterior; post.: posterior; ave.: average; BLQ: below the limit of quantification. Scale of B and C; and of D and E are equivalent.

The average time between completion of HIVEC and excision of bladder tissue was 27 min. The mean concentration of DOX in the bladder wall was 13.6 μg/*g* ± 4.67 μg/g (Figure 7(A)). No difference was detected between DOX concentrations in the anterior and posterior bladder wall. DOX accumulation appeared greatest in connective tissue between inner longitudinal and circular muscle fibers (Figure 7(B,C)), and around blood vessels (Figure 7(D,E)). No MMC was detected (lower limit of detection: 20 ng/mL) 30 min after HIVEC. The 30-min time point for tissue drug measurement reflects the approximate time needed to obtain full-thickness bladder wall samples by laparotomy. DOX and MMC were undetectable in the bladder, heart, kidney, spleen, rectum, lung, and liver at 28 days.

### Pharmacokinetics

**Figure 8.**
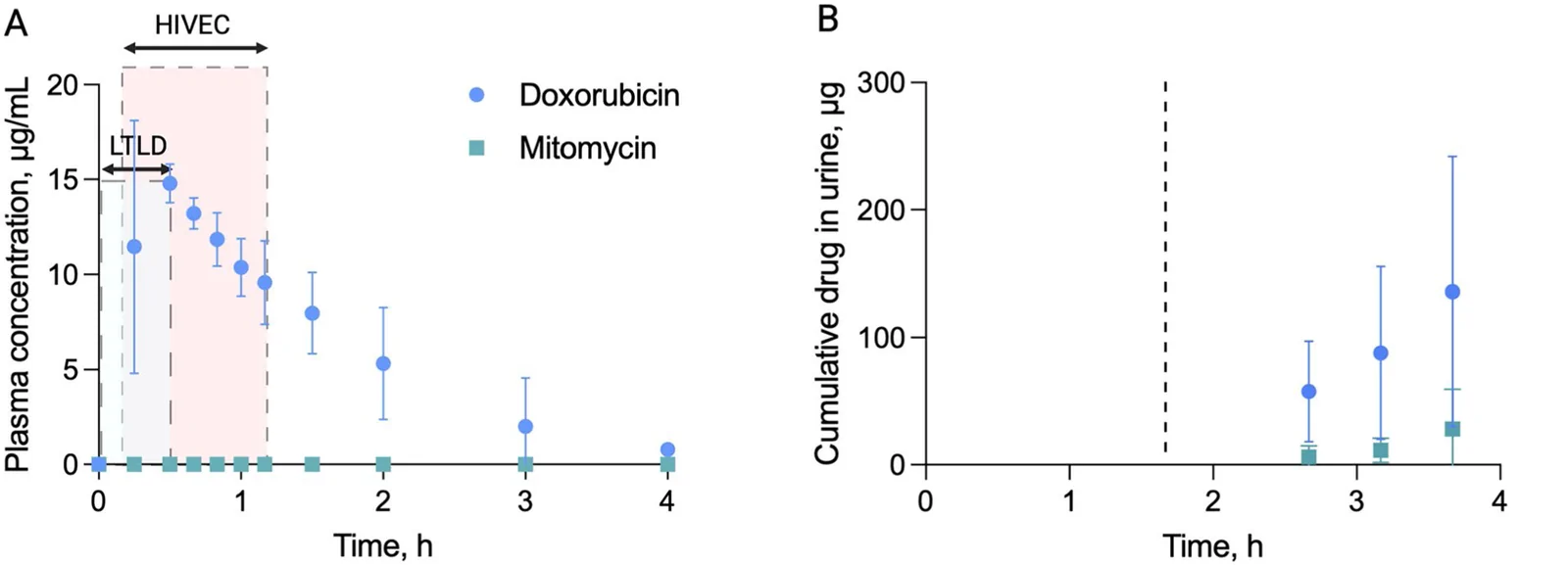
Plasma pharmacokinetics: A) plot of plasma DOX and MMC concentrations during and after LTLD infusion (0.7 mg/kg, blue box with long, dashed outline) and HIVEC (40 mg, red box with short, dashed outline). HIVEC began 10 min after the start of LTLD infusion (*t* = 0) and continued for one hour. B) Cumulative amount of DOX and MMC in urine. Dashed line in B indicates the time of initial bladder emptying prior to the start of urine accumulation in the bladder and collection (30 min after HIVEC following bladder wall sampling).

Plasma pharmacokinetic parameters are presented in Table 1. Plasma concentrations of DOX and MMC up to 4 h after initiation of LTLD infusion are shown in Figure 8(A). MMC was not detected in systemic circulation; however, both DOX and MMC were detected in urine (Figure 8(B)).

## Discussion

This study demonstrates that combination therapy with intravenous LTLD and hyperthermic intravesical chemotherapy (HIVEC) is both technically feasible and well tolerated in a healthy swine model. No treatment-related adverse events, systemic toxicity, or weight loss were observed over the 28-day post-treatment period. DOX accumulated preferentially in the bladder wall in all layers, particularly within connective tissues and near vasculature. These findings suggest that this treatment may be safe for further investigation as a potential bladder-sparing therapy for NMIBC.

Computational modeling identified that starting LTLD infusion 10 min before HIVEC maximized doxorubicin deposition in the bladder wall. Although longer HIVEC durations may be feasible if tolerated, only modest increases in drug deposition are expected, as plasma LTLD concentrations decline to approximately half of C_max_ by the end of 1-h HIVEC in this swine model. Since DOX delivery to heated tissues with LTLD is positively correlated with plasma DOX concentrations, extending heating beyond 1 h is unlikely to substantially enhance tissue uptake [22]. The mean DOX concentration measured (13.6 ± 4.67 μg/g) in the bladder wall exceeded our previous study using temperature-sensitive liposomes and bladder-localized hyperthermia [14], possibly reflecting more precise temperature control and uniform heating by the commercial bladder recirculation system used for HIVEC in this study. This finding is also comparable to intravenous 1.2-dipalmitoyl-sn-glycero-3-phosphodiglycerol (DPPG_2_) thermosensitive liposomal DOX combined with radiofrequency-induced bladder heating in swine, given the lower dose (0.7 mg/kg vs. 1 mg/kg), 30-min post-HIVEC washout period, and full-thickness bladder wall drug measurements used in our study [15]. The greater AUC_0–1h_ (659.22 vs 443.4 ng/μL*min) despite the lower dose, and longer elimination half-life (45 vs. 25 min) of LTLD also suggest a larger window for delivery of DOX that could be exploited by prolongation of hyperthermia. Potential advantages of the bladder recirculation system compared to radiofrequency-based hyperthermia systems include its ease of clinical integration and operation, compact size and portability, and low cost.

The pharmacokinetic profile of LTLD demonstrated first order plasma elimination kinetics and partial urinary excretion of DOX. The 45 min elimination half-life for LTLD was longer than reported for the DPPG_2_-containing formulation [15], but reflects substantially more rapid clearance in swine compared to humans [23], possibly due to greater uptake of liposomes by the mononuclear phagocyte system in swine [24]. The AUC of LTLD was greater than that reported for systemic administration of free DOX in swine, assuming AUC dose proportionality [25], owing to the greater circulation longevity of LTLD.

Systemic absorption of MMC was not detected in blood above 20 ng/mL, consistent with previous reports in both animal and human studies for various intravesical MMC doses and dwell times [26–28]. MMC was detected in urine, likely reflecting diffusion of low concentrations of MMC from the bladder wall during the 2.5-h sampling period. Synergistic cytotoxic effects between DOX and MMC have been demonstrated in patient-derived bladder cancer cell cultures *in vitro* [29], supporting the rationale for combining these agents during HIVEC.

The absence of measurable MMC in the bladder wall after HIVEC may be due to washout of drug via blood flow during the 25–30-min delay between HIVEC completion and surgical tissue collection, or via diffusion into saline instilled in the bladder during specimen excision. Wientjes et al. reported a rapid decline in bladder wall MMC concentrations following removal of intravesical MMC prior to ligation of bladder vasculature, with a half-life of 4 min and undetectable levels after 30 min [7]. Methodological differences may also account for our findings including the use of full-thickness bladder wall samples for drug quantification, while other studies used mucosal or superficial biopsies where MMC concentrations are highest [26,30]. Additionally, some studies used longer dwell times [6,7], higher MMC concentrations [31], or primed the recirculation system with extra MMC [26]. Finally, MMC uptake is known to be greater in bladder tumors than in healthy tissue, which may limit detection in this healthy swine model.

MMC uptake into the bladder wall is affected by dwell time and intravesical concentration, which is determined by the total volume used to dissolve the drug and prime the recirculation system [9,26,31]. Clinical HIVEC protocols vary, with MMC doses ranging from 40–120 mg and circulating fluid concentrations between 0.5–1.3 mg/mL. Comparative studies are lacking, but a swine study showed that hyperthermia with a 60-min dwell time and 80 mg MMC (1.3 mg/mL) maximized mucosal MMC levels, with minimal systemic absorption similar to normothermic intravesical MMC [26]. Doses of 40, 80, and 120 mg have shown comparable safety and tolerability in BCG-unresponsive patients [32–34].

Despite its widespread use in various cancers, systemic DOX is associated with dose-dependent myelosuppression and cardiotoxicity, including a 1–5% risk of cardiomyopathy at cumulative doses exceeding 550 mg/m^2^ [35]. Targeted delivery of DOX via LTLD combined with localized mild bladder hyperthermia may reduce toxicity to non-target organs. A phase I dose-escalation study of LTLD (ThermoDox®) with radiofrequency ablation in patients with liver lesions established a maximum tolerated dose of 50 mg/m^2^. In our study, an LTLD dose equivalent to approximately 40 mg/m^2^ in swine was well tolerated, with no evidence of myelosuppression at 28 days.

This study has several limitations. Uptake of intravesical chemotherapy is typically higher in bladder tumors than in healthy tissue, which could result in greater local and systemic drug concentrations than observed in healthy swine used in this study [7]. Nevertheless, these concentrations are still expected to remain below the threshold for MMC systemic toxicity [9]. Temperatures within the bladder wall were not directly measured; however, perfusion differences between the healthy bladders evaluated in this study and bladder tumors may alter heat transfer and, in turn, influence LTLD drug release and uptake during HIVEC. The sample size was small and quantification of DOX in blood and tissue did not distinguish between encapsulated and free DOX. Transient myelosuppression may not have been observed because blood was not sampled between 7–10 days after treatment, the expected timeframe for toxicity from conventional intravenous doxorubicin. However, owing to the heat-triggered, targeted release of LTLD, systemic exposure to free DOX is lower, and off-target toxicities are expected to be reduced. No clinical symptoms of toxicity were observed, and baseline blood counts were confirmed at day 28. The delay between the end of HIVEC and bladder tissue collection may have reduced the ability to detect MMC in the bladder wall. Finally, potential differences in liposome clearance between swine and humans should be considered when interpreting pharmacokinetic data.

## Conclusions

Combination therapy with intravenous LTLD and MMC HIVEC was both feasible and well tolerated, making it a potentially attractive treatment for patients with BCG-unresponsive non-muscle invasive bladder cancer.

## Figures and Tables

**Table 1.** Non-compartmental pharmacokinetic analysis of doxorubicin for 30 min intravenous infusion of LTLD at a dose of 0.7 mg/kg combined with HIVEC.

Parameter	Doxorubicin

C_max_, μg/mL	16.4 ± 3.46
T_max_, h	0.5 (0.25–0.5)
AUC_0−∞_, μg/mL · h	23.2 ± 5.50
t_1/2 elimination_, h	0.743
CL, mL/h	2574±702

Data are expressed as means with standard deviation except T_max_, which is expressed as median and range.

## Data Availability

The datasets generated during and/or analyzed during the current study are available from the corresponding author on reasonable request.
